# Mechanical and Durability Analysis of Fly Ash Based Geopolymer with Various Compositions for Rigid Pavement Applications

**DOI:** 10.3390/ma15103458

**Published:** 2022-05-11

**Authors:** Muhammad Faheem Mohd Tahir, Mohd Mustafa Al Bakri Abdullah, Shayfull Zamree Abd Rahim, Mohd Rosli Mohd Hasan, Andrei Victor Sandu, Petrica Vizureanu, Che Mohd Ruzaidi Ghazali, Aeslina Abdul Kadir

**Affiliations:** 1 Centre of Excellence Geopolymer and Green Technology, (CEGeoGTech), Universiti Malaysia Perlis, Perlis 01000, Malaysia; shayfull@unimap.edu.my; 2 Faculty of Chemical Engineering Technology, Universiti Malaysia Perlis, Perlis 01000, Malaysia; 3 Faculty of Mechanical Engineering Technology, Universiti Malaysia Perlis, Perlis 01000, Malaysia; 4 School of Civil Engineering, Universiti Sains Malaysia, Pulau Pinang 14300, Malaysia; cerosli@usm.my; 5 Faculty of Material Science and Engineering, Gheorghe Asachi Technical University of Iasi, 41 D. Mangeron St., 700050 Iasi, Romania; 6 Romanian Inventors Forum, St. P. Movila 3, 700089 Iasi, Romania; 7 Technical Sciences Academy of Romania, Dacia Blvd 26, 030167 Bucharest, Romania; 8 Faculty of Ocean Engineering Technology and Informatic, Universiti Malaysia Terengganu, Kuala Terengganu 21030, Malaysia; ruzaidi@umt.edu.my; 9 Faculty of Civil Engineering and Built Environment, Universiti Tun Hussein Onn Malaysia, Parit Raja 86400, Malaysia; aeslina@uthm.edu.my

**Keywords:** rigid pavement, fly ash based geopolymer, compressive strength, acid resistance

## Abstract

Ordinary Portland cement (OPC) is a conventional material used to construct rigid pavement that emits large amounts of carbon dioxide (CO_2_) during its manufacturing process, which is bad for the environment. It is also claimed that OPC is susceptible to acid attack, which increases the maintenance cost of rigid pavement. Therefore, a fly ash based geopolymer is proposed as a material for rigid pavement application as it releases lesser amounts of CO_2_ during the synthesis process and has higher acid resistance compared to OPC. This current study optimizes the formulation to produce fly ash based geopolymer with the highest compressive strength. In addition, the durability of fly ash based geopolymer concrete and OPC concrete in an acidic environment is also determined and compared. The results show that the optimum value of sodium hydroxide concentration, the ratio of sodium silicate to sodium hydroxide, and the ratio of solid-to-liquid for fly ash based geopolymer are 10 M, 2.0, and 2.5, respectively, with a maximum compressive strength of 47 MPa. The results also highlight that the durability of fly ash based geopolymer is higher than that of OPC concrete, indicating that fly ash based geopolymer is a better material for rigid pavement applications, with a percentage of compressive strength loss of 7.38% to 21.94% for OPC concrete. This current study contributes to the field of knowledge by providing a reference for future development of fly ash based geopolymer for rigid pavement applications.

## 1. Introduction

Pavements are an essential part of our life as we use them as roads, highways, driveways, and parking lots. Pavements are an important engineering structure for trading, commerce, and defence because they provide a smooth, flat, and durable all-weather travelling surface for a variety of vehicles and users. The construction of pavements will continue to be a major industry for both developing and developed countries. Pavements can be divided into two types, asphalt (or flexible) pavements and concrete (or rigid) pavements, which are composed of different layers [1]. Binder or surface, base, subbase, and subgrade are the layers from top to bottom. The primary function of pavements is to distribute load from the surface to the sub-grade, allowing them to withstand the load applied by vehicles or users without deforming excessively.

The initial construction cost of flexible pavement is lower when compared to rigid pavement, as bituminous surfacing materials are cheap, and construction of flexible pavement does not require extra reinforcements such as joints and steel bars. Furthermore, thermal stress cannot be induced, as flexible pavement is free to contract and relax, thus it is more resistant to temperature changes [2]. Rutting, which is a permanent deformation or rut depth along the wheel load part on the movable asphalt surface over time, is a major distress mode for flexible pavement [3]. In addition, it is more susceptible to oil stains and chemical damages. However, among all types of road pavements, rigid pavements have the greatest advantages in terms of durability and ability to maintain shape under continuous traffic and harsh environmental conditions. Although rigid pavements are generally expensive, they require less maintenance and have a good design life [4]. However, the installation and maintenance process of rigid pavements, such as grouting and subgrade treatment are expensive. Table 1 summarises the major benefits and drawbacks of each type of pavements previously discussed.

Both flexible and rigid pavements are vital to the economic and social development of a country because they contribute to other sectors, namely, education, health, employment, and social services. The mechanical and durability characteristics of the road surface are equally important for providing resistance to degradation processes during the expected life of the road surface. The durability of concrete mainly depends on the characteristics of the pore structure of the pavement and the size of the cracks. Water penetration, chloride ions, CO_2_, acids (including chlorides), and sulphates in the pavements are all related to its durability [2,3,4].

Rigid pavements are constructed by placing concrete slab on a stabilized subgrade, or base, or subbase if extra structural support is required. Most of the rigid pavements are made of Portland cement concrete (PCC), which has a high rigidity, flexural strength, and modulus of elasticity, allowing the load to be evenly distributed over a larger area of soil and providing a large portion of the structural capacity [5]. However, the production of cement is an energy consuming and carbon-intensive process, and it is a main contributor to global CO_2_ emission [6,7,8,9]. The cement industry is one of the largest sources of CO_2_, where production of one ton of cement will release approximately 900 kg of carbon dioxide into the environment, causing global warming and depletion of the ozone layer [7]. Aside from emission of large amounts of CO_2_, there are also other disadvantages found in rigid pavement constructed by ordinary Portland cement (OPC). PCC has a low resistance to chemical attack. Acid corrosion resistance of OPC is rather poor because of the nature of high pH and porous matrix. Acid can react with CH and C-S-H gel in cement concrete to form non-gelling or water-soluble substances, resulting in the destruction of the concrete. To overcome these disadvantages, fly ash based geopolymer is introduced as an alternative to OPC.

Fly ash based geopolymer can be produced by activating fly ash, which is rich in silica, and alumina material with alkaline solution [10], and it is defined as a binding phase comprising aluminosilicate gel where aluminium and silicon are linked into a three-dimensional tetrahedral gel framework [11]. It has several advantages compared to OPC. First, fly ash based geopolymer has higher durability than OPC, as it has denser layer of aluminosilicate gel, causing it to have low permeability and preventing it from corrosion by acid [9]. It is also proven that production of fly ash based geopolymer can emit 5 to 6 times less CO_2_ when compared to OPC, as high temperature calcination is not required [12]. Fly ash based geopolymer also has higher workability than OPC due to its spherical shape [13].

In current studies [10,11,12,13,14], the potential role of geopolymers as a substitute for OPC in pavement production is being explored because of its significant positive impact on the environment, society, and economy. However, its performance as a rigid pavement material is limited, and there is no compelling evidence that it could replace typical OPC concrete firm pavements. Although many studies have been done on fly ash based geopolymer [9,10,11,12,13,14,15,16], the studies with regard to the optimization of mix design remain scarce. This study is an initiative in realizing that mechanical and durability properties are crucial aspects in applying fly ash based geopolymer as rigid pavements. In this research, the optimum ratio of fly ash/alkaline activator, sodium silicate/sodium hydroxide, and concentration of sodium hydroxide to yield fly ash based geopolymer concrete that has optimum strength for newly constructed rigid pavements are investigated. In addition, the durability of fly ash based geopolymer and OPC rigid concrete pavement in acidic environment are investigated and compared.

## 2. Materials and Method

### 2.1. Materials

In this study, class F fly ash based on ASTM C618 [17] was used as the raw material and source material for the geopolymer binder, aluminosilicates. Fly ash was collected from Manjung Power Station, located in Lumut, Perak, Malaysia. Sodium hydroxide and sodium silicate were used to produce an alkaline activator. Sodium hydroxide (NaOH) pellets were obtained from Formosa Plastic Corporation, Taiwan, and sodium silicate (Na_2_SiO_3_) solution was supplied by South Pacific Chemical Industries Sdn. Bhd. (SPCI), Perai, Penang, Malaysia. Coarse aggregates, which consist of crushed stone with particle size larger than 5 mm, and fine aggregates, which consist of sand obtained from the river, were used for making the concrete mixes. Five percent sulphuric acid solution was used to test the acid resistance of the concrete mixes.

### 2.2. Methodology

**Figure 1:**
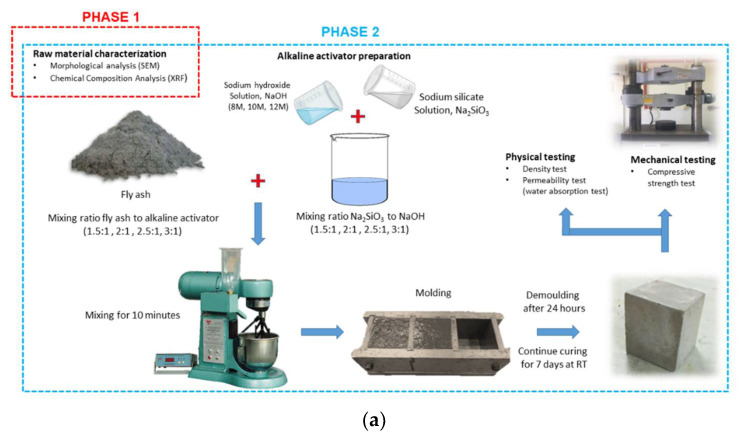

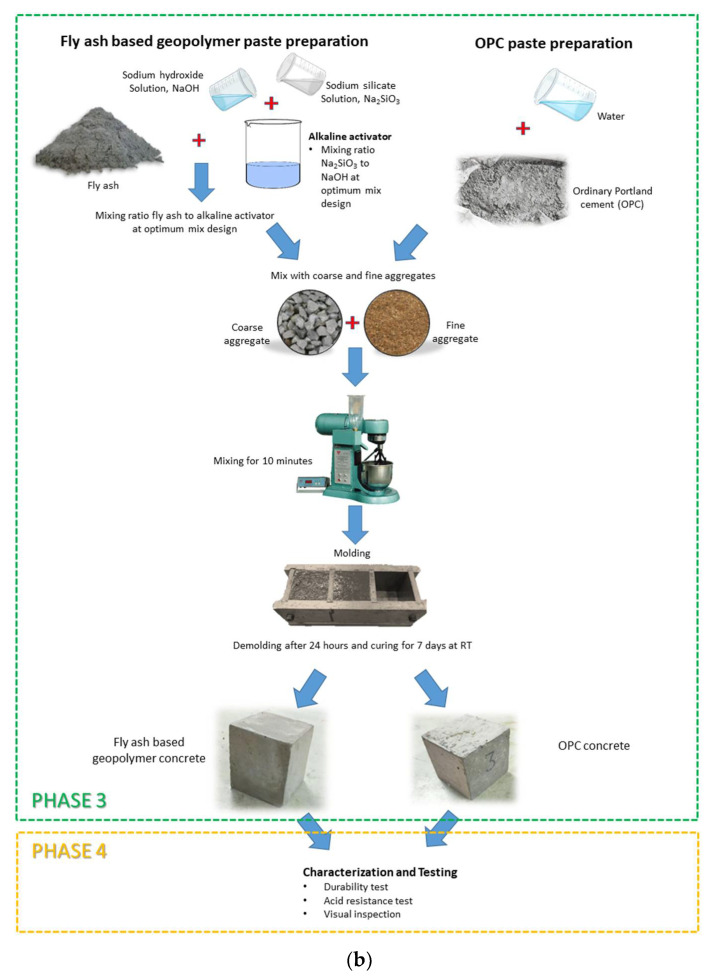
(**a**) Flow chart for fly ash based rigid pavements application process (Phase 1 and Phase 2). (**b**) Flow chart for fly ash based rigid pavement application process. (Phase 3 and Phase 4).

The investigation of fly ash based geopolymer for rigid pavement application is divided into four phases. Based on Figure 1a, Phase 1 is raw material characterization. In this stage, the morphology of class F fly ash is determined using scanning electron microscopy (SEM), JEOL Ltd., Tokyo, Japan, and the chemical composition of class F fly ash is determined using X-ray fluorescence (XRF), Bruker Malaysia Sdn. Bhd., Penang, Malaysia. The microstructure analysis of fly ash is in the form of powder that is spread onto a carbon tape. The samples are then coated with palladium by using Auto Fine Coater JEOL JFC 1600 model prior to testing. Phase 2 is the synthesis of fly ash based geopolymer using different mix designs, and determination of density, water absorption, and compressive strength of the samples.

Fly ash based geopolymer is prepared by mixing class F fly ash with the alkaline activator. To achieve good solid–liquid homogeneity, sodium hydroxide was mixed with sodium silicate for a few minutes before fly ash is added, according to ASTM C305 [18]. After fly ash has been added, a scraper was used to tamp into the mould several times to release the air trapped in the geopolymer paste. Another way to release the trapped air is to vibrate the mould. The solution was mixed quickly to prevent it from curing before casting. In this study, the effects of concentration of NaOH, ratio of solid-to-liquid, and ratio of sodium silicate to sodium hydroxide on density, water absorption, and compressive strength were investigated. The best mix design that produces fly ash based geopolymer with optimum compressive strength required for rigid pavement application was determined.

Based on Figure 1b, Phase 3 includes the synthesis of OPC concrete and fly ash based geopolymer concrete using the optimum mix design determined from Phase 2. Both types of concrete were made using the same mix design. There was no addition of water during synthesis of geopolymer concrete as it has already obtained water from the alkaline solution. Table 2 shows the ratio of material based on M40 mix design.

The fourth phase is the durability testing of fly ash based geopolymer concrete and OPC concrete exposed in an acidic environment in accordance with ASTM C267 [19]. An acid immersion test was carried out, and the percentage of compressive strength loss and weight loss was calculated to determine the durability of both concretes after immersing in 5% sulphuric acid. Next, visual inspection was done on both concrete samples.

## 3. Result and Discussion

### 3.1. Chemical Composition of Fly Ash

The most abundant chemical content in fly ash is silicon dioxide (SiO_2_), which accounts for 52.11% according to the XRF analysis. Silica in silicon dioxide is the source material for the geopolymer, as the product of geopolymer synthesis is an aluminosilicate gel that requires silica to be formed [13]. The gel undergoes further geopolymerisation by eliminating water and converting it into strong and durable material with excellent mechanical strength [20]. The second most abundant chemical content is alumina (Al_2_O_3_), which accounts for 23.59% in fly ash. Al_2_O_3_ is also the main component of geopolymer, as it is a polymeric chain made up of silica and alumina that shares the oxygen ion. Al_2_O_3_ and SiO_2_ react with alkaline activators, namely, NaOH and Na_2_SiO_3_, in the geopolymerisation process.

Iron oxide (Fe_2_O_3_) is one of the main chemical constituents, which accounts for 7.39% in fly ash. It contributes to the dark colour of fly ash. Aside from appearance, it also increases the specific gravity value of fly ash. The percentage of calcium oxide (CaO) content in fly ash is 2.61%. During the geopolymerisation process, calcium oxide forms CSH and CASH gels within geopolymer binder, and these gels are responsible for the increase in strength and reduction in setting time [21]. The calcium content in the raw material is considered as low, resulting in the geopolymer having longer setting time. Loss of ignition (LOI) content in fly ash is 9.59%, which is considered high, as ASTM C618 [12] prescribes a maximum of LOI content of 6% by weight. LOI quantifies the total content of unburned coal residue. High LOI causes detrimental effects that include high water demand, leading to high porosity and reducing the compressive strength of prepared geopolymers. There were also other chemical compounds found in fly ash. Table 3 tabulates the chemical composition of fly ash collected from Manjung Power Station, which is located in Lumut, Perak, Malaysia.

For class F fly ash, ASTM C618 [17] specifies a total composition of silicon oxide (SiO_2_), alumina (Al_2_O_3_), and iron oxide (Fe_2_O_3_) of at least 70%, and less than 10% calcium oxide (CaO). As the total content of SiO_2_, Al_2_O_3_, and Fe_2_O_3_ is 83.09% and CaO content is 2.61%, it is classified as Class F fly ash.

### 3.2. Morphological Analysis of Fly Ash

**Figure 2:**
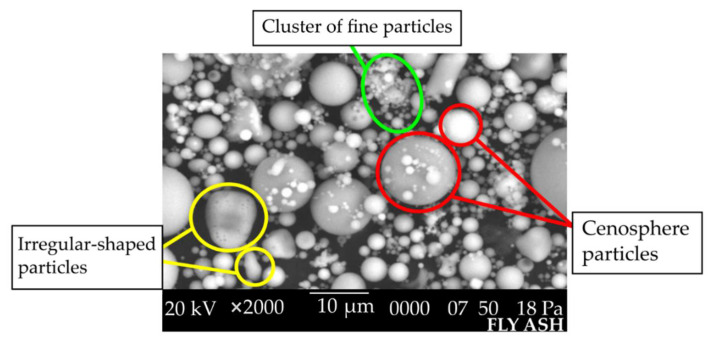
SEM micrograph of fly ash.

Using ImageJ Ver. 1.5 software, the fly ash particle was shown to have a mean particle size of 4.543 µm with a minimum of 1.903 µm and a maximum of 11.534 µm. It can be seen that fly ash consists of series of cenosphere particles of different sizes. Cenospheres are hollow spherical particles filled with gas that is mostly CO_2_ and nitrogen (N_2_). When undergoing alkaline attack, the wall of cenospheres will dissolve and release Si and Al ions as supported by Rahman [22]. There were also some irregularly shaped particles observed. This is because the original mineral in coal is not sufficiently fired. The coarse particles that appear to consist of a cluster of fine particles are possibly formed by aggregation of molten aluminosilicate droplets during cooling, where fine droplets could minimize surface free energy. Figure 2 illustrates the morphological characteristics of Class F fly ash using SEM analysis.

Geopolymer synthesis starts with the leaching of silica and alumina on the surface of fly ash. The spherical shape and fine size of fly ash particles allows a large surface area to be exposed to the alkaline activator and increases the dissolution rate. The higher the surface area and the higher the number of particles, the better the aluminosilicate gel formation. The fine particle size of fly ash also helps to increase compressive strength and accelerate initial setting time of geopolymer. The spherical shape also causes the sliding between particles to be easier, resulting in high flowability geopolymer paste. Therefore, the amount of liquid required to produce geopolymer paste would be lower. This is crucial because the less water used, the lower the porosity, as previous studies have shown [22,23,24,25].

### 3.3. Optimization of Fly Ash Based Geopolymer for Rigid Pavement Application

#### 3.3.1. Effect of Molarity of NaOH on Density of Fly Ash Based Geopolymer

**Figure 3:**
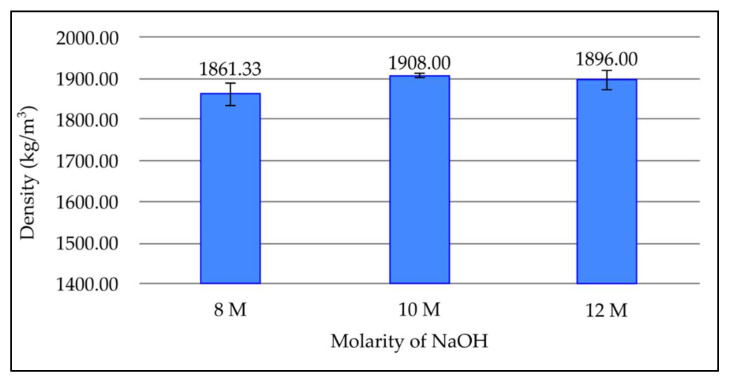
Density of fly ash based geopolymer with different molarities of NaOH.

Figure 3 illustrates the density values of geopolymer paste with different molarities of sodium hydroxides after 7 days curing period at room temperature. Based on Figure 3, the density increases when NaOH molarity increases from 8 M to 10 M, which is from 1861.33 kg/m^3^ to 1908.00 kg/m^3^, respectively. However, the density then decreases to 1896.00 kg/m^3^ when NaOH molarity is further increased to 12 M. The density of geopolymer is the lowest at 8 M, which is 1861.33 kg/m^3^, and highest at 10 M, which is 1908.00 kg/m^3^.

It can be seen that NaOH concentration does not have a significant effect on density. The density of fly ash based geopolymer increased slightly when NaOH concentration increases to 10 M. This is because at this concentration, there are enough Na^+^ and OH^−^ ions to complete the geopolymerisation process and form dense aluminosilicate gel. At high concentration, fly ash undergoes a greater dissolution process from the leaching of silica and alumina [24]. However, the density reduces as molarity increased to 12 M. Due to fast setting, increasing the NaOH concentration may result in paste with lower density due to a mixing problem. The higher concentration of NaOH limits the flow of geopolymer and reduces the setting time. This causes poor compaction and increases the porosity of the geopolymer, resulting in low density.

#### 3.3.2. Effect of Molarity of NaOH on Water Absorption of Fly Ash Based Geopolymer

**Figure 4:**
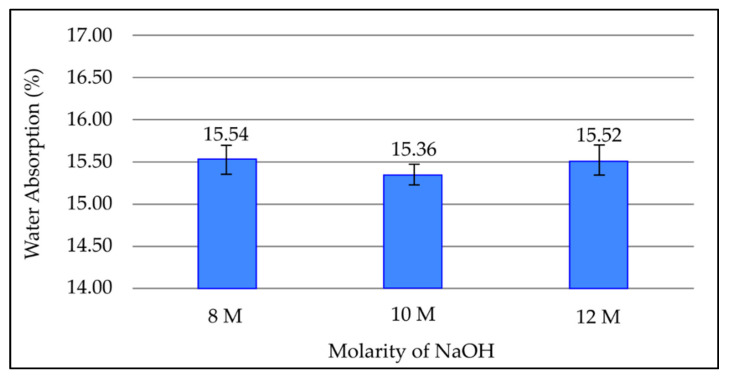
Water absorption of fly ash based geopolymer with different molarities of NaOH.

Figure 4 shows the effect of different molarities of sodium hydroxides on water absorption in geopolymer paste after 7 days curing period at room temperature. Based on Figure 4, the water absorption percentage decreases when molarity increases to 10 M, which is 15.54% at 8 M and 15.36% at 10 M. Furthermore, the water absorption percentage increases to 15.52% when the molarity further increased to 12 M. The maximum water absorption percentage is recorded at a molarity of 8 M, which is 15.54%, whereas the minimum value is recorded at 10 M, which is 15.36%.

The effect of NaOH concentration on water absorption of fly ash based geopolymer is not significant. Water absorption of the geopolymer decreases a little when molarity increases to 10 M. This is because as concentration of NaOH solution increases, the leaching of silica and alumina ion increases as well. Sufficient amounts of Si^4+^ and Al^3+^ ions allow more aluminosilicate gel to form and reduce the pores in the geopolymer, thus reducing water absorption of the material. Water absorption then increases again at a molarity of 12 M. This is due to excess concentration of sodium hydroxide that causes unreactive alkali solution, which weakens the binding of sodium components in the geopolymer structure. Similar results were obtained from another study where 10 M NaOH was found to be the optimum value for synthesis of fly ash based geopolymer [26].

#### 3.3.3. Effect of Molarity of NaOH on Compressive Strength of Fly Ash Based Geopolymer

**Figure 5:**
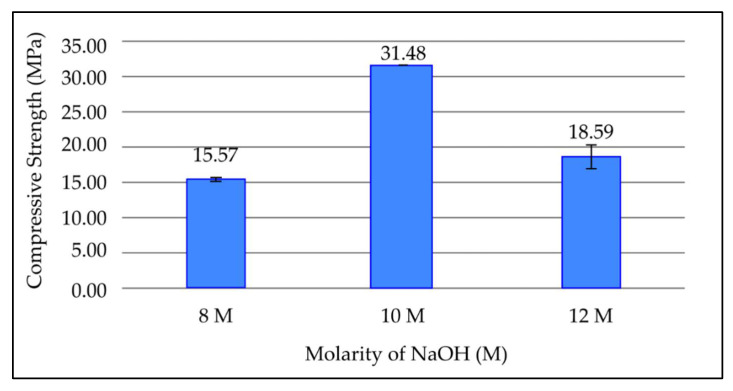
Compressive strength of fly ash based geopolymer with different molarities of NaOH.

Figure 5 shows the effect of various sodium hydroxide molarities on compressive strength in geopolymer paste after 7 days curing period at room temperature. Based on Figure 5, the compressive strength of geopolymer increases when molarity of NaOH increases with values of 15.57 MPa and 31.48 MPa for molarities of 8 M and 10 M, respectively. The compressive strength then dropped to 18.59 MPa at a molarity of 12 M. The highest value of compressive strength was obtained at a molarity of 10 M, which is 31.48 MPa.

The strength of the geopolymer paste is affected by the molarity of NaOH because it affects the dissolution of Si^4+^ and Al^3+^ ions in fly ash particles. The compressive strength increases when molarity of NaOH increases due to the increase of OH^-^ concentration, which accelerates the dissolution and hydrolysis processes. During the dissolution process, the leaching of Si^4+^ an Al^3+^ ion enhances the formation of aluminosilicate gel and contributes to high strength in the geopolymer. In addition, the amount of NaOH is high enough to maintain the charge balance for the substitution of tetrahedral Si by Al [27]. However, the compressive strength drops when the molarity of NaOH is further increased to 12 M. This is because excess hydroxide ion concentration causes aluminosilicate gel precipitation at an early age. The precipitation prevents further leaching of Si^4+^ and Al^3+^ ions, therefore lowering the compressive strength. This is supported by studies that found that 10 M is the optimum molarity of NaOH for the synthesis of fly ash based geopolymer [28].

#### 3.3.4. Effect of NaOH to Na_2_SiO_3_ (SS/SH) Ratio on Density of Fly Ash Based Geopolymer

**Figure 6:**
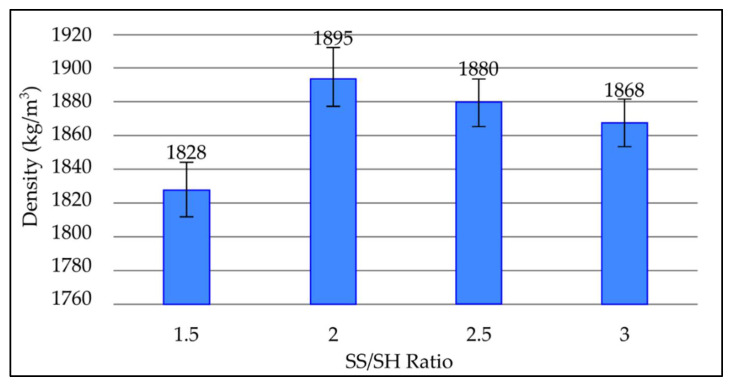
Density of fly ash based geopolymer with different SS/SH ratios.

Figure 6 depicts the change in density of geopolymer samples with various SS/SH ratios after a 7-day room temperature curing period. It can be seen that the density increases until the SS/SH ratio reaches 2. However, as the SS/SH ratio increases beyond 2, the density decreases gradually to 1880 kg/m^3^ and 1858 kg/m^3^ at SS/SH ratios of 2.5 and 3, respectively. The lowest density obtained was 1828 kg/m^3^ at an SS/SH ratio of 1.5. Density is the highest at an SS/SH ratio of 2, which is 1895 kg/m^3^.

As the SS/SH ratio increases to 2, the high silica content encourages the formation of N-A-S-H (sodium aluminosilicate) gel, which provides good compact structure, subsequently increasing the density of the geopolymer paste. Thus, the highest value of density is obtained at an SS/SH ratio of 2. The drop in density after the SS/SH ratio exceeds 2 is due to the excess sodium silicate, which hinders water evaporation and structure formation [29]. In addition, the excessive sodium silicate content retards the geopolymerisation process, as formation of Al-Si phase precipitation prevents interaction between reacting material and the alkaline activator [30,31]. These factors increase the porosity and reduce density of the geopolymer.

#### 3.3.5. Effect of NaOH to Na_2_SiO_3_ (SS/SH) Ratio on Water Absorption of Fly Ash Based Geopolymer

**Figure 7:**
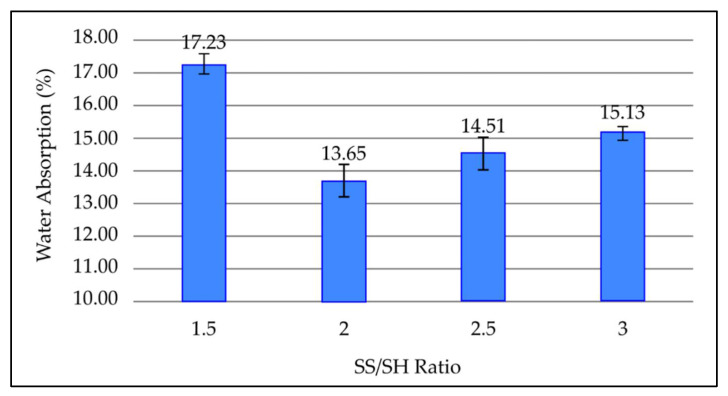
Water absorption percentage of fly ash based geopolymer with different SS/SH ratios.

Figure 7 portrays the effect of various SS/SH ratios on the water absorption percentage of a fly ash-based geopolymer after 7 days curing period at room temperature. Based on Figure 7, the water absorption percentage decreases from 17.23% at an SS/SH ratio of 1.5 to 13.65% at an SS/SH ratio of 2. The water absorption percentage then increases when the SS/SH ratio further increases from 2. The highest water absorption percentage recorded was at an SS/SH ratio of 1.5, which is 17.23%, whereas the lowest value is obtained at an SS/SH ratio of 2, which is 13.65%.

By referring to Figure 7, the water absorption of geopolymer paste decreases as the SS/SH ratio increases to 2. As the SS/SH ratio increases, a higher ratio of sodium silicate increases the formation of N-A-S-H gel and reduces the pores in the geopolymer paste, consequently reducing water absorption. The water absorption then increases when the SS/SH ratio further increases from 2. This is because the coagulation of silica happens due to excessive amounts of sodium silicate. This coagulation separates the aluminosilicate source from the alkali activators and prevents further geopolymerisation, resulting in increased porosity and higher water absorption percentage. These findings are consistent with previous research [22,23,32].

#### 3.3.6. Effect of NaOH to Na_2_SiO_3_ (SS/SH) Ratio on Water Absorption of Fly Ash Based Geopolymer

**Figure 8:**
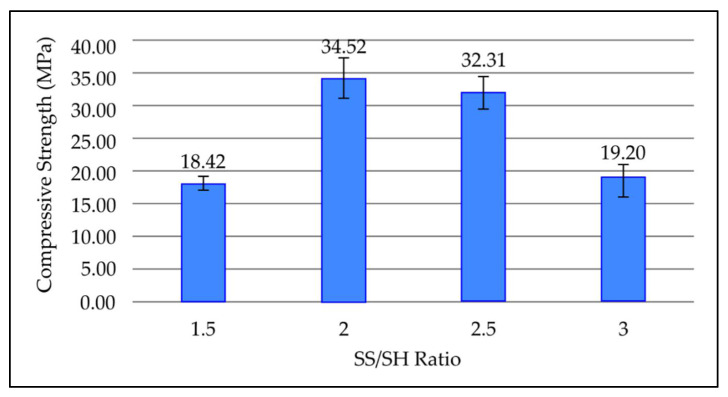
Compressive strength of fly ash based geopolymer with different SS/SH ratios.

Figure 8 shows the compressive strength values for various SS/SH ratios in geopolymer paste after 7 days curing time at room temperature. Figure 8 highlights that when the SS/SH ratio increases to 2.5 and 3, the compressive strength of geopolymer steadily decreases. The maximum compressive is obtained at an SS/SH ratio of 2, which is 34.52 MPa, and the strength reduced to 32.31 MPa and 19.20 MPa at SS/SH ratios of 2.5 and 3, respectively. The lowest compressive strength obtained is 18.42 MPa at an SS/SH ratio of 1.5.

Compressive strength increases when the SS/SH ratio reaches 2. As the amount of NaSiO_3_ increases, the ratio of Si/Al increases. In comparison to aluminium, more silicon is required in the structure of geopolymers such as poly (sialate), poly (sialate-siloxo), and poly (sialate-disiloxo). In addition, high silica content promotes the formation of Si-O-Si bond, which makes materials stronger [33,34,35]. However, further increases in the SS/SH ratio will result in strength loss, because extra soluble silicate species hinders the reaction between silicate and aluminate species. Ultimately, the dissolution did not occur or was reduced, causing the material to lose strength and the majority of the silica to remain unreacted.

#### 3.3.7. Effect of Solid-to-Liquid (S/L) Ratio on Density of Fly Ash Based Geopolymer

**Figure 9:**
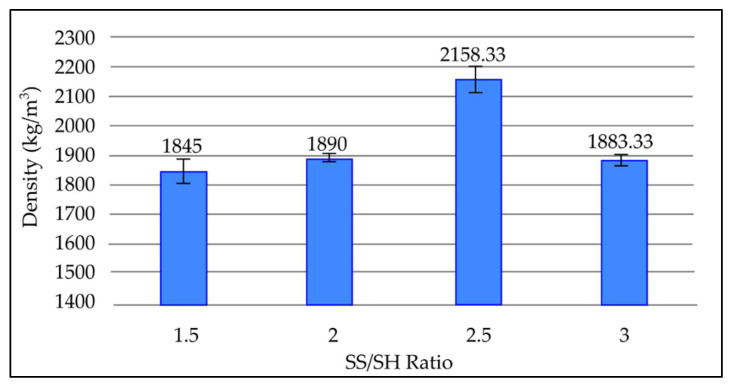
Density of fly ash based geopolymer with different S/L ratios.

Figure 9 shows how adjusting the S/L ratio affects the density of fly ash-based geopolymer that has been cured for 7 days at room temperature. When the S/L ratio reaches 2.5, the density rises, as seen in Figure 9. However, when the S/L ratio increases to 3, the density reduces. The highest density value is found at an S/L ratio of 2.5, which is 2158.33 kg/m^3^, whereas the lowest value is found at an S/L ratio of 1.5, which is 1845 kg/m^3^.

Density increases as the S/L ratio increases until it reaches 2.5. This is due to the reduction of the alkaline activator content. High content of alkaline activator results in excessive OH^-^ left in the system, which weakens the geopolymer structure. In addition, excess sodium content can form sodium carbonate by atmospheric carbonation and may disrupt the polymerization process [36,37]. Therefore, reduction of the alkaline activator allows denser geopolymer structure to form and increases the density of the geopolymer paste. However, density reduces as the S/L ratio increases further to 3, as high solid content reduces workability. The mixing becomes undesirable and undergoes a compaction problem during the moulding process. This increase in porosity reduces the density of geopolymer. Research conducted by Sing et al. [38] showed similar results that stated that an S/L ratio of 2.5 produced geopolymer with the highest density.

#### 3.3.8. Effect of Solid-to-Liquid (S/L) Ratio on Water Absorption of Fly Ash Based Geopolymer

**Figure 10:**
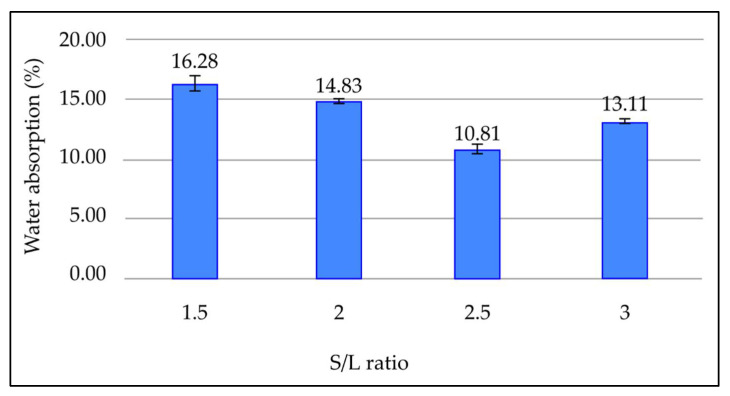
Water absorption percentage of fly ash based geopolymer with different S/L ratios.

Figure 10 presents the effect of S/L ratio on water absorption of the geopolymer after being cured for 7 days at room temperature. As shown in Figure 10, the water absorption percentage of geopolymer decreases as the S/L ratio rises. When the S/L ratio exceeds 2.5, however, the percentage of water absorbed again increases. The minimum water absorption percentage is 10.81% at an S/L ratio of 2.5, whereas the highest water absorption percentage is 16.28% at an S/L ratio of 1.5.

The water absorption percentage decreases as the S/L ratio increases to 2.5. This is due to reduction of excessive OH^-^ contributed by the alkaline solution, which weakens the geopolymer structure. Furthermore, lowering the alkaline content prevents excess sodium from forming sodium carbonate, which stymies the geopolymerisation process. Therefore, as alkaline solution reduces and fly ash content increases to a certain extent, denser geopolymer structure could be formed, resulting in geopolymer with less porosity, and thus reduced water absorption. Water absorption percentage increases when the S/L ratio increases beyond 2.5. These results are in line with those reported by previous researchers [38]. This is because increasing the S/L ratio will increase the setting time. This reduces workability and causes difficulty in mixing and compaction, consequently resulting in more pore formation and increased water absorption percentage in geopolymer paste.

#### 3.3.9. Effect of Solid-to-Liquid (S/L) Ratio on Compressive Strength of Fly Ash Based Geopolymer

**Figure 11:**
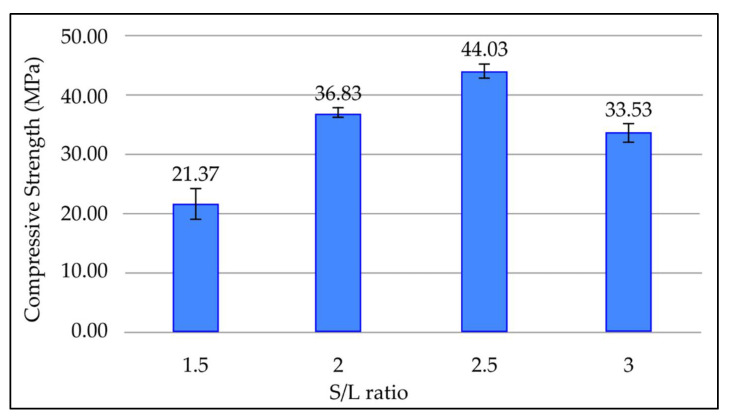
Compressive strength of fly ash based geopolymer with different S/L ratios.

Figure 11 shows the effect of various S/L ratios on compressive strength in geopolymer paste after 7 days curing time at room temperature. Figure 11 highlights that when the S/L ratio approaches 2.5, the compressive strength of geopolymer rises. Compressive strength reduces as the S/L ratio exceeds 2.5. The highest compressive strength is 44.03 MPa at an S/L ratio of 2.5, whereas the minimum compressive strength is 21.37 MPa at an S/L ratio of 1.5.

Compressive strength of geopolymer paste increases as the S/L ratio increases to a certain point. As the S/L ratio increases, the rate of intermolecular contact between precursor material and alkaline activator increases as the volume of fluid medium reduces. This increases the rate of dissolution of aluminosilicate material and therefore causes the compressive strength of geopolymer to rise. Further increase of the S/L ratio will reduce the compressive strength of geopolymer. This is due to insufficient alkaline activator to activate the aluminosilicate source materials, causing less reaction product to form and reducing compressive strength. Moreover, the presence of a high amount of unreacted fly ash increases the roughness of the matrix and reduces the compressive strength of the material [39,40].

### 3.4. Durability Analysis

#### 3.4.1. Acid Resistance Test on Concrete

**Figure 12:**
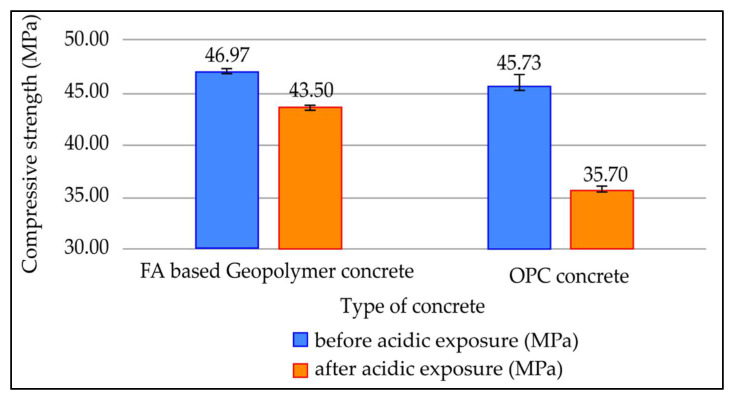
Compressive strength of concrete before and after acidic exposure for 28 days.

Figure 12 highlights the compressive strength of fly ash based geopolymer concrete and OPC concrete before and after immersing in 5% concentration of sulphuric acid for 28 days. Both concrete mixes were cured for 7 days prior to the acid immersion. According to Figure 12, the compressive strengths of fly ash based geopolymer concrete before and after exposure to acidic solution are 46.97 MPa and 43.50 MPa, respectively, whereas the compressive strengths of OPC concrete before and after exposure to acidic solution are 45.73 MPa and 35.70 MPa, respectively. The percentage of compressive strength loss for fly ash based geopolymer concrete is 7.38%, and the percentage of compressive strength loss for OPC concrete is 21.94%. The percentage of compressive strength loss for OPC concrete is higher compared to fly ash based geopolymer concrete.

After 28 days of acidic exposure, OPC concrete had a larger percentage of compressive strength loss than fly ash based geopolymer. This is because OPC is vulnerable to acid assault due to its high calcium concentration, which raises the Ca/Si ratio. The presence of free calcium causes the cement paste to deteriorate and the creation of gypsum and ettringite, which can lead to a loss of mechanical performance. As fly ash-based geopolymers have low calcium content, the reaction produces less ettringite and gypsum after sulphuric acid exposure, resulting in less mechanical degradation. Furthermore, due to the creation of a thick layer of aluminosilicate gel, fly ash-based geopolymers have lesser permeability, resulting in a longer decalcification process and less strength loss [41,42].

**Figure 13:**
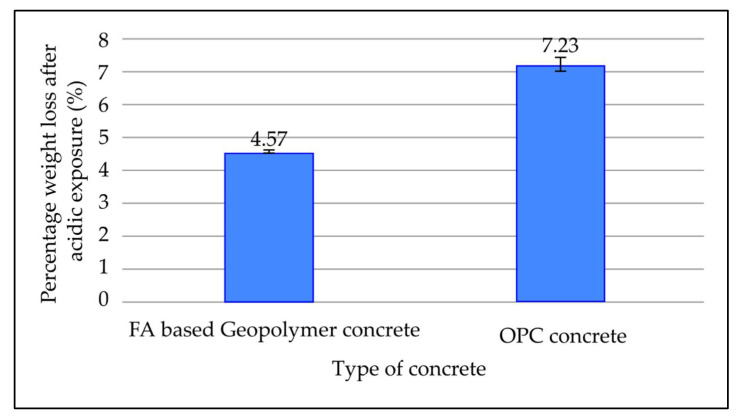
Percentage weight loss of concrete mixes after acidic exposure for 28 days.

Figure 13 illustrates the weight loss of fly ash-based geopolymer concrete and OPC concrete after being cured for 7 days at room temperature and then immersed in a 5% sulphuric acid solution for 28 days. Based on the figure, OPC concrete loses 7.23% of its weight, whereas fly ash-based geopolymer loses 4.57% following acidic exposure. When compared to geopolymer concrete made with fly ash, OPC concrete lost more weight following acid immersion.

OPC concrete exhibits high weight loss due to its higher calcium content. Its weight losses are mainly attributed to the reaction between calcium hydroxide, which is Ca(OH)_2_, and sulphuric acid, which causes tensile stress and increases crack and delamination of concrete. Due to the reaction between sulphuric acid and calcium hydroxide, high calcium content in OPC concrete causes more gypsum and ettringite to form, leading to expansion, dimensional instability, cracking, spalling, softening, and mass loss. The low weight loss of fly ash based geopolymer is due to its lower content of calcium. The low calcium content causes lower formation of gypsum and ettringite [43]. The low weight loss of fly ash based geopolymer concrete is also due to its higher resistance to water penetration compared to OPC concrete. The pores in geopolymer are filled with alumino-silcates, which lower its permeability. There are more pores persisting in the OPC concrete to enable the hydration of the cement [44]. Due to low permeability, a lower amount of acid will penetrate into the structure to erode the interior.

#### 3.4.2. Appearance of Exposed Concrete

**Figure 14:**
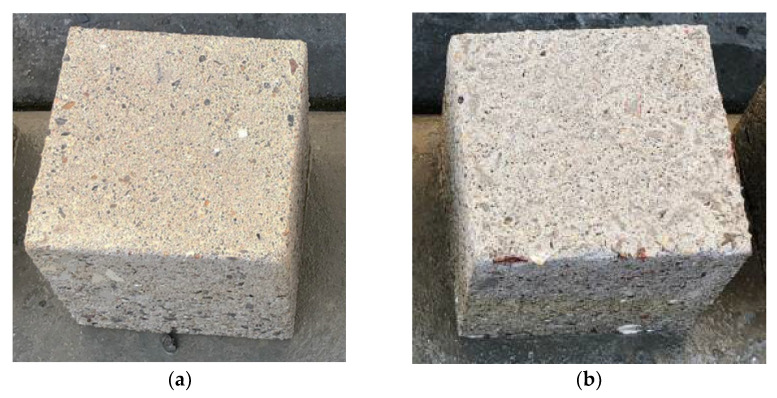
Concrete sample after acidic exposure for 28 days: (**a**) geopolymer, (**b**) OPC.

**Figure 15:**
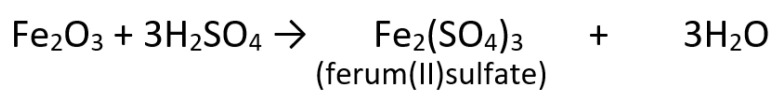
Chemical reaction of ferum(II)oxide with sulfuric acid.

Figure 14a shows the appearance of exposed fly ash based geopolymer concrete under an optical microscope after it is immersed in 5% sulphuric acid for 28 days, whereas Figure 14b illustrates the appearance of OPC concrete after immersion in 5% sulphuric acid for 28 days. Based on Figure 14a,b, it can be seen that the aggregates in OPC concrete are more visible after acidic exposure compared to aggregates from the surface of fly ash based geopolymer concrete. For OPC concrete, the surface erosions can be easily observed, whereas moderate surface erosions were observed in fly ash based geopolymer concrete. OPC concrete undergoes more deterioration in sulphuric acid solution compared to fly ash based geopolymer. Furthermore, the surface colour of OPC concrete changed from grey to white due to the existence of gypsum, which is white in colour, whereas the colour of fly ash geopolymer changed from grey to slightly brown due to the reaction of iron (II) oxide with sulphuric acid to produce iron (II) sulphate [45]. The yellowish line at the surface of geopolymer is most probably the result of the reaction of ferum (II)oxide with sulfuric acid and produced ferum (II) sulfate, as shown in Figure 15.

**Figure 16:**
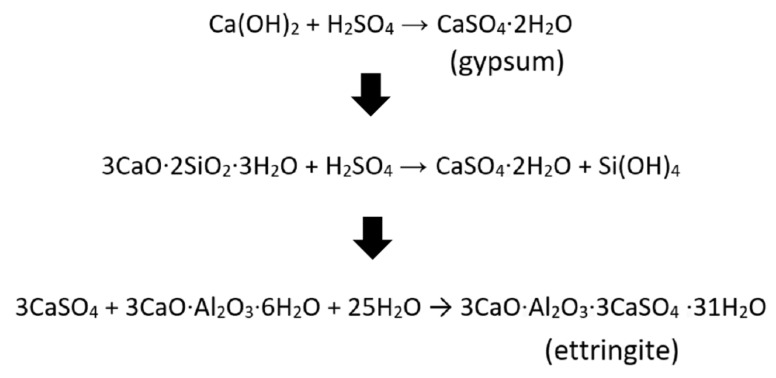
Chemical reaction of OPC with sulfuric acid.

OPC concrete is more susceptible to acid attack due to the presence of hydration products, namely Ca(OH)_2_. The Ca(OH)_2_ on the surface of OPC concrete is consumed by the reaction with acid and turned into gypsum, which is soft and porous and causes the surface of OPC concrete to deteriorate. Subsequently, gypsum would undergo distractive reaction with tricalcium aluminates within the cement matrix, resulting in the formation of calcium sulphoaluminate (ettringite), which has large volume and causes expansive deterioration mechanism. The reaction of OPC with sulfuric acid is presented in Figure 16.

Fly ash-based geopolymers do not contain hydration products; they produce N-A-S-H gel, which is acid resistant and has less surface deterioration. Fly ash also has low calcium content, resulting in the formation of less gypsum and ettringite that cause expansive deterioration mechanism. The change in colour of geopolymer from grey to slightly brown is due to the reaction of iron (II) oxide with sulphuric acid to produce iron (II) sulphate, as supported by previous researchers [46].

## 4. Conclusions and Future Work

In this study, the best formulation for synthesis of fly ash based geopolymer with highest compressive strength in terms of concentration of NaOH, SS/SH ratio, and S/L ratio was determined. In addition, the durability of both fly ash based geopolymer and OPC concrete in an acid environment were tested and compared. Based on the results obtained from the analysis and experimental data, the following conclusions can be drawn:

- The concentration of NaOH of 12 M, SS/SH ratio of 2.0, and S/L ratio of 2.5 are the optimum parameters to synthesize fly ash based geopolymer.
- The optimum ratio of sodium silicate to sodium hydroxide (SS/SH ratio) for synthesis of fly ash based geopolymer is 2.0, as geopolymer with an SS/SH ratio of 2.0 produced the best result compared to other the SS/SH ratios of 1.5, 2.5, and 3.0.
- The optimum solid to liquid (S/L) ratio for fly ash based geopolymer is 2.5. This S/L ratio yields fly ash based geopolymer with the highest compressive strength compared to ratios (1.5, 2.0, and 3.0).
- As the concentration of NaOH increases to 12 M, the OH^−^ concentration increases, which accelerates the dissolution and hydrolysis processes. As the ratio of SS/SH increases, the Si/Al ratio increases and favours the formation of strong bonds. As the S/L ratio increases to 2.5, the rate of intermolecular contact between precursor material and alkaline activator increases, consequently increasing the rate of dissolution of aluminosilicate material.
- The use of this formulation produces fly ash based geopolymer with compressive strength of 47 MPa, which exceeds the minimum compressive strength required for rigid pavement application based on Standard Specification for Road Work by Jabatan Kerja Raya (JKR).
- It is also found that the percentage of compressive strength loss and weight loss of fly ash based geopolymer concrete is lower compared to that of OPC concrete after acid immersion. Fly ash based geopolymer is less susceptible to acid attack due to low calcium content and low permeability. OPC concrete suffers from high surface erosion due to the presence of hydration product Ca(OH)_2_. The acid reacts with Ca(OH)_2_ to form gypsum and ettringite, which are soft and porous.

Because the mechanical properties and durability of fly ash based geopolymer concrete are higher compared to OPC based concrete, it is highly recommended to use this material for rigid pavement application, as it suffers from less deterioration under acid attack compared to OPC concrete, and this could lower the maintenance cost required. This demonstrates that fly-based geopolymer is more suitable for use in rigid pavement applications due to its high mechanical performance and durability, which could result in lower rigid pavement maintenance costs. It is beneficial to expand future research on the study of using fly ash based geopolymer as rigid pavements in terms of quality, effect of environment temperature, and long-term durability when exposed to harmful environmental conditions.

## Figures and Tables

**Table 1:** Basic characteristics of flexible and rigid road pavements [4].

Type of Pavement	Advantages	Disadvantages
Flexible Pavements	Can be used in the pre-construction stage Simple maintenance; can be opened and repaired Inexpensive materials Can easily repair frost swelling and sedimentation Prevent ice glaze formation Shorter management time means shorter traffic and business interruption No connectors required during installation	The service life is shorter than that of rigid pavements Frequent maintenance is required, which increases costs Easily damaged by oil stains and other chemicals The edges are weak, so curb structures or edges are needed
Rigid Pavements	Longer service life Less maintenance Allows future asphalt resurfacing Allows for wider load distribution with fewer basic and sub-basic requirements Can be installed on low-quality and high-quality soil Does not require extra trimming work or firm edges of curbs Oil spills and chemical damage resistant	Expensive initial installation Expensive maintenance cost Riding quality is low and very rough Concrete shrinkage and expansion under various conditions require support joints

**Table 2:** Mix design for grade M40 design.

Cement/Binder	Fine Aggregates	Coarse Aggregates	Water/Cement Ratio
1	1.84	2.65	0.4

**Table 3:** Fly ash chemical composition by X-ray fluorescence (XRF).

Chemical Composition	Percentage (%)
SiO_2_	52.11
Al_2_O_3_	23.59
Fe_2_O_3_	7.39
TiO_2_	0.88
CaO	2.61
MgO	0.78
Na_2_O	0.42
K_2_O	0.80
P_2_O_5_	1.31
SO_3_	0.49
MnO	0.03
LOI	9.59

## Data Availability

Not applicable.
